# Supplementary material for: Mie-coupled bound guided states in nanowire geometric superlattices
Source: Nat Commun. 2018 Jul 17;9:2781. doi: 10.1038/s41467-018-05224-2 (PMC6050327; doi:10.1038/s41467-018-05224-2)
Supplement: Supplementary file 1 — Supplementary Information [file 41467_2018_5224_MOESM1_ESM.pdf]

Supplementary Information for:

Mie-Coupled Bound Guided States in Nanowire Geometric Superlattices

Seokhyoung Kim[1], Kyoung-Ho Kim[1,2], David J. Hill[1], Hong-Gyu Park[2], and James F. Cahoon[1*]*

Supplementary Information includes:

- Supplementary Figures 1-11
- Supplementary Tables 1-2
- Supplementary Movie 1-4 captions

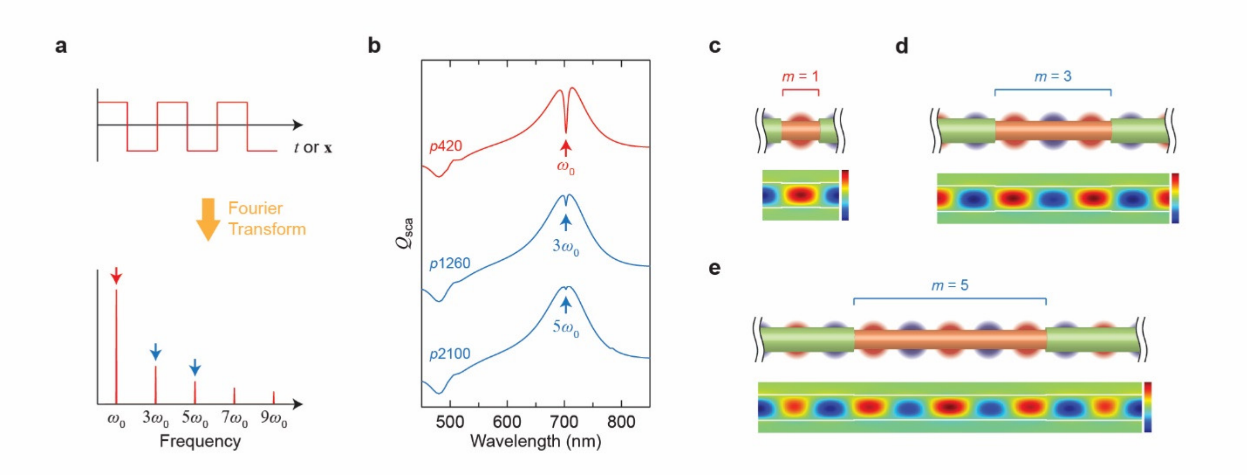

Supplementary Figure 1 | Fourier selection of BGS by higher harmonics. a, A square wave representing a GSL structure and its Fourier series that consists of odd-numbered harmonics of the fundamental frequency. **b,** Q_{sca} spectrum of NW GSLs with d = 140 nm and e = 135 nm of three different values of p = 420 nm (top), p = 1260 nm (middle) and p = 2100 nm (bottom). Pitch was varied to tailor higher harmonic interactions with a Mie resonance fixed at the same wavelength. **c-e,** Schematic and calculated magnetic field profiles for the GSLs in panel b at the dip, showing a BGS mode with m = 1 (**c**), m = 3 (**d**) and m = 5 (**e**) antinodes confined at each segment.

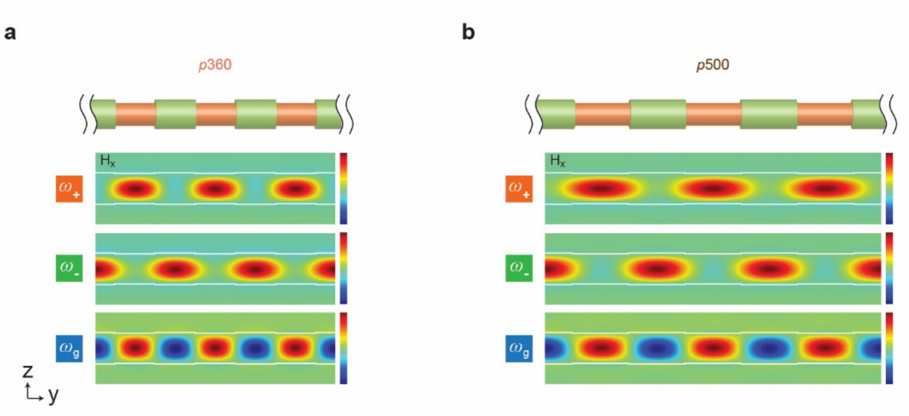

Supplementary Figure 2 | Calculated Magnetic Field Profiles of a GSL with varying pitch. a-b, Schematic and calculated magnetic field profiles for a GSL with p = 360 nm (**a**) and p = 500 nm (**b**) from Figure 1c, showing coupled TM_{11} dipolar modes appearing in small diameter regions (ω_+, top) and large diameter regions (ω_-, middle), and a standing wave as a superposition of counter-propagating HE_{11} guided modes (ω_g, bottom).

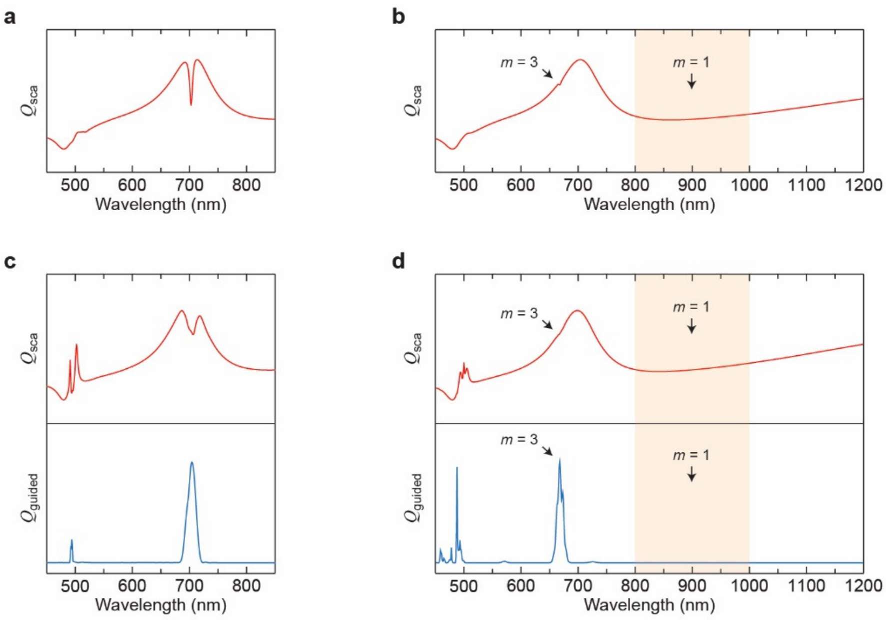

Supplementary Figure 3 | Disappearance of BGS coupling at long pitch values outside the Mie resonance envelope. a-b, Simulated Q_{sca} spectra with d = 140 nm and e = 135 nm for an on-resonance p = 420 nm (**a**) and an off-resonance p = 1000 nm (**b**) NW GSL under plane wave illumination. A scattering dip is not observed for p = 1000 nm in the shaded region of the spectrum where the first-order (m = 1) BGS is expected, but the third-order (m = 3) mode appears at ~668 nm. **c-d,** Simulated Q_{sca} spectra (upper graphs) and Q_{guided} spectra (lower graphs) for an on-resonance p = 420 nm (**c**) and an off-resonance p = 1000 nm (**d**) GSL-WG under illumination with a Gaussian beam. For the off-resonance structure (panel **d**), no guided power is observed in the shaded region of the spectrum where the first-order (m = 1) BGS is expected, but guided power from the third-order (m = 3) mode appears at ~668 nm.

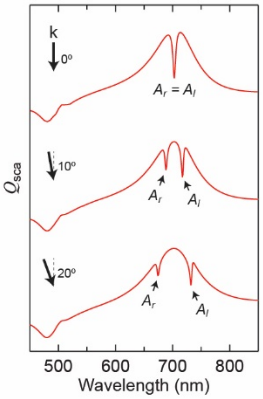

Supplementary Figure 4 | Incident angle dependence of BGS coupling. Simulated Q_{sca} spectra of a NW GSL with d = 140 nm, e = 135 nm, and p = 420 nm under plane wave illumination at 0º (upper), 10º (middle), and 20º (bottom) with respect to the normal, showing a splitting of the BGS into primarily right-propagating (A_r) and left-propagating (A_l) BGS modes.

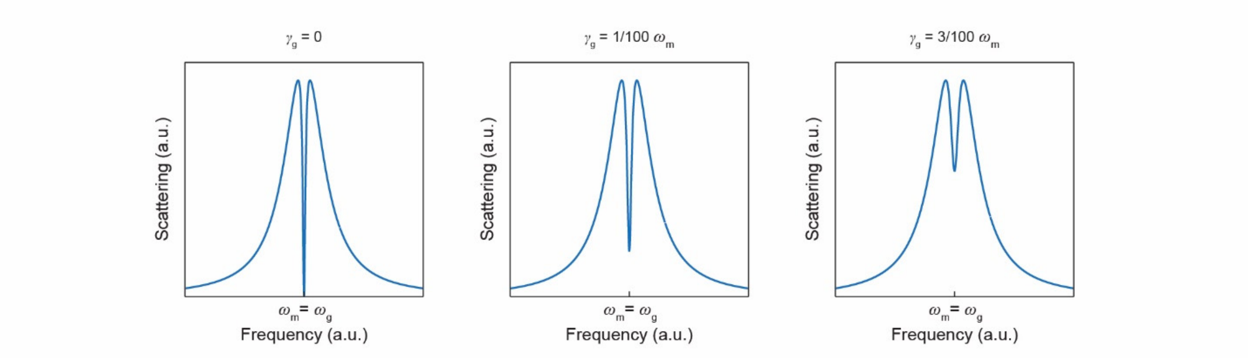

Supplementary Figure 5 | Schematic plots of Mie-BGS coupling with and without γ_g. Schematic scattering plots for $\omega_m = \omega_g$ when γ_g is zero (left), 1/100 ω_m (middle) and 3/100 ω_m (right).

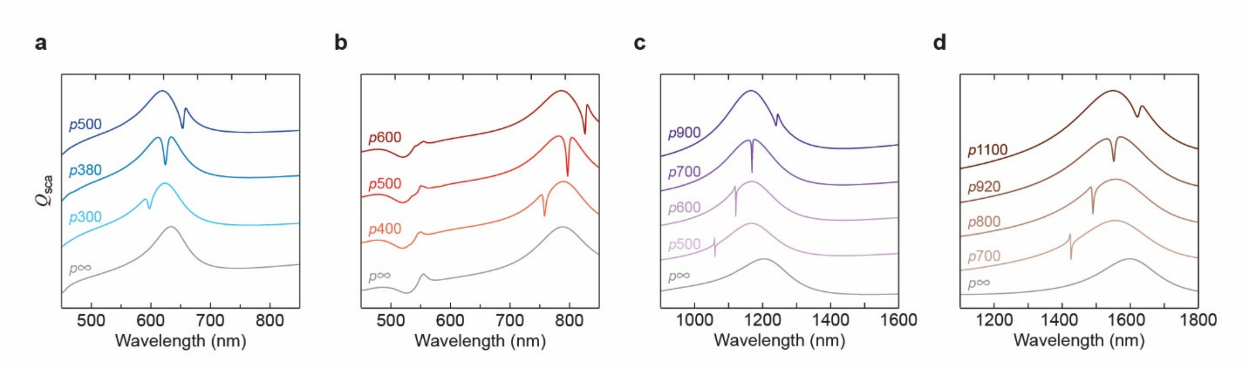

Supplementary Figure 6 | Diameter dependence and spectral tunability of Mie-BGS coupling. Simulated Q_{sca} spectra of GSLs of selected pitches with d = 120 nm, e = 115 nm (**a**), d = 160 nm, e = 155 nm (**b**), d = 250 nm, e = 245 nm (**c**), and d = 340 nm, e = 330 nm (**d**).

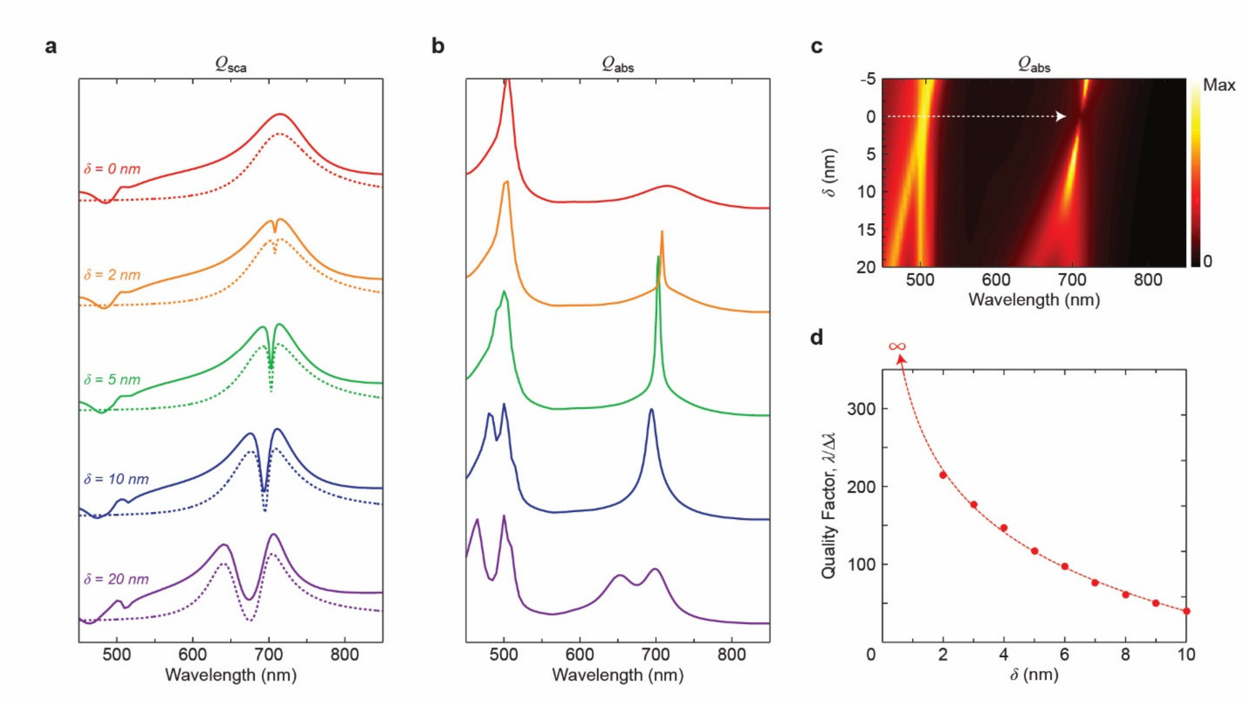

Supplementary Figure 7 | Effect of modulation depth on bound character of the BGS. a, Numerically calculated Q_{sca} spectra (solid lines) and analytical fit by TCMT (dotted lines) for a NW GSL with d =140 nm, p = 420 nm and varying δ of 0, 2, 5, 10, and 20 nm. δ = 0 nm corresponds to a NW with uniform diameter. Parameters for TCMT spectra are provided in Table S2. **b,** Numerically calculated Q_{abs} spectra of GSLs from panel **a**, showing a broad background peak for δ = 0 nm and appearance of a high-quality factor absorption peak for non-zero δ that eventually bifurcates into two peaks at δ > 15 nm. **c,** Two-dimensional Q_{sca} heat map with vertical and horizontal axes for δ and wavelength, respectively, showing a vanishing point at δ = 0 nm that corresponds to a completely bound guided state (BGS). **d,** Quality factor ($\lambda/\Delta\lambda$) calculated from the fit parameters (Supplementary Table 2) used panel **b** plotted against δ, which extrapolates to infinity as δ approaches zero.

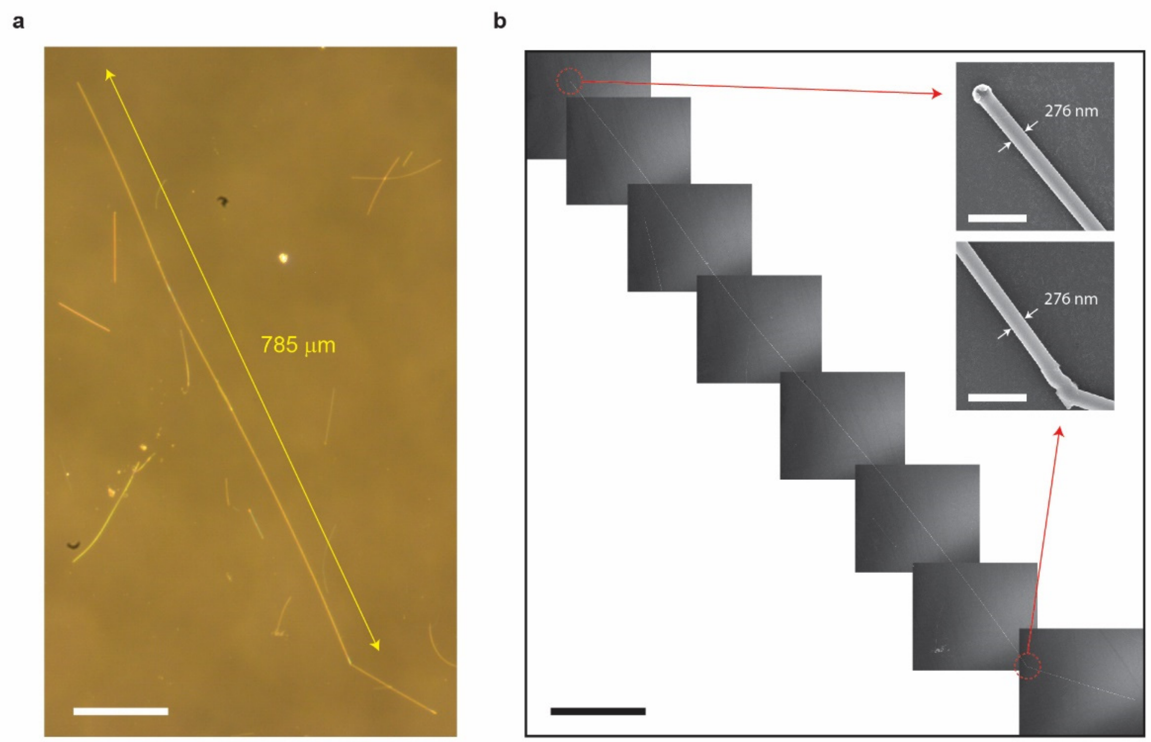

Supplementary Figure 8 | NW with length of nearly 1 mm with no variation in diameter. a-b, Optical (**a**) and SEM (**b**) images of a NW exhibiting no diameter variation over nearly 1 mm; scale bars, 100 µm. Insets: Magnified views at each end of a straight portion of the NW; scale bars, 1 µm.

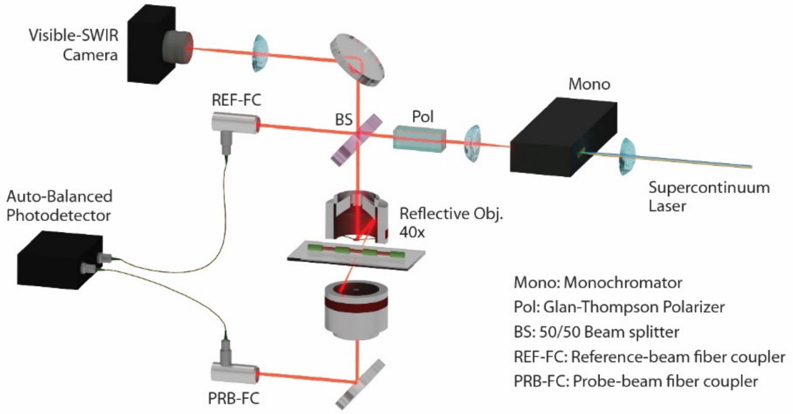

Supplementary Figure 9 | Instrumental setup. Laser microscope for polarization-resolved bright-field extinction measurements.

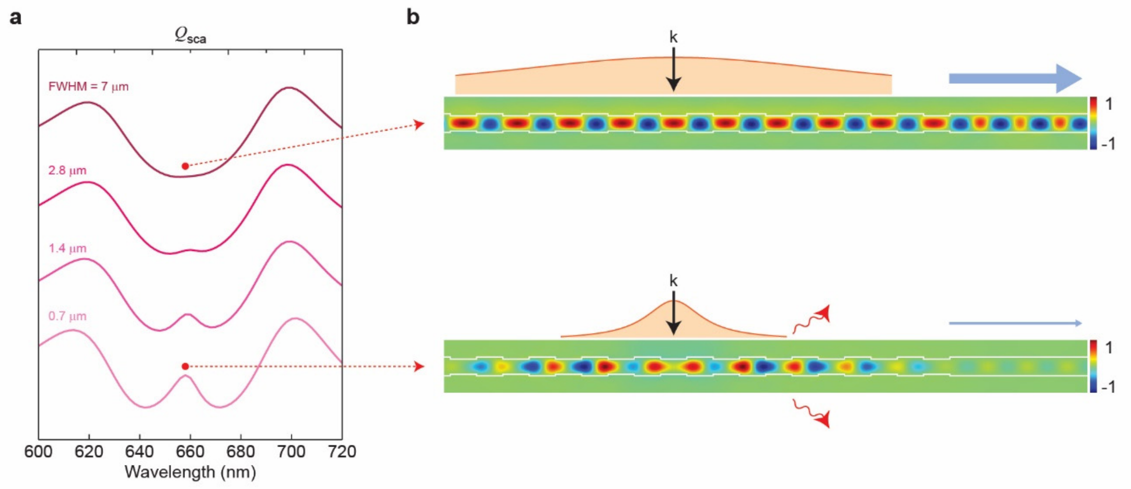

Supplementary Figure 10 | Effect of Gaussian beam width. a, Q_{sca} spectra around the scattering dip region of a GSL with d = 140 nm, e = 115 nm and p = 400 nm under focused Gaussian illumination of varying width. Each spectrum is labeled with its Gaussian full-width at half maximum. Absorptive loss is artificially turned off to only account for scattering characteristics. **b,** Calculated magnetic field snapshots at marked locations from panel **a** and Supplementary Movie 3-4, showing coherent coupling to the BGS under a large beam (top) and radiative loss under a small beam (bottom).

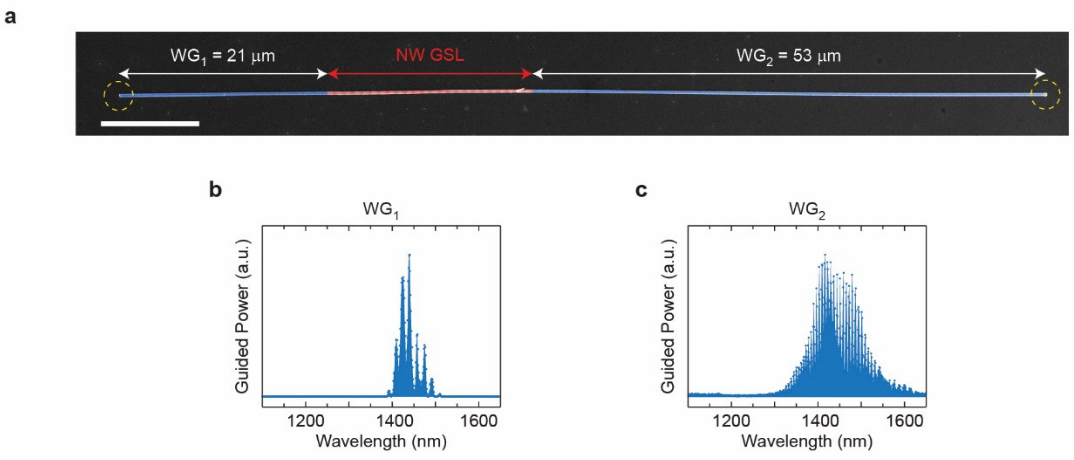

Supplementary Figure 11 | Fabry-Perot cavity free spectral ranges in WGs of different lengths. a, A NW GSL attached to WGs at both ends in lengths of 21 and 53 μm; scale bar, 10 μm. **b-c,** Experimental guided spectra collected from WG_1 (**b**) and WG_2 (**c**).

Supplementary Table 1 | Parameters used in the TCMT for fitting Q_{sca} spectra in Figure 1c

	λ_m (nm)	λ_g (nm)	ω_m (THz)	ω_g (THz)	ω_c (THz)	γ_m (THz)	γ_g (THz)
$p\infty$	700	1000	2691	1884	-[a]	177	-[a]
$p360$	700	680	2691	2770	24	179	5.4
$p420$	700	703	2691	2679	30	179	5.4
$p500$	700	726	2691	2595	30	179	4.5

[a] Any arbitrary value can be chosen.

Supplementary Table 2 | Parameters used in the TCMT for fitting Q_{sca} spectra in Supplementary Figure 7

δ (nm)	λ_m (nm)	λ_g (nm)	ω_m (THz)	ω_g (THz)	ω_c (THz)	γ_m (THz)	γ_g (THz)
0	711	-[a]	-[a]	-[a]	0	177	-[a]
2	707	708	2664	2661	12	178	5.3
5	700	703	2691	2679	30	179	5.4
10	687	695	2742	2710	55	183	5.5
20	665	675	2833	2791	129	189	5.7

[a] Any arbitrary value can be chosen.
