# Supplementary material for: Mie-coupled bound guided states in nanowire geometric superlattices
Source: Nat Commun. 2018 Jul 17;9:2781. doi: 10.1038/s41467-018-05224-2 (PMC6050327; doi:10.1038/s41467-018-05224-2)
Supplement: Supplementary file 3 — Description of Additional Supplementary Files [file 41467_2018_5224_MOESM3_ESM.pdf]

Description of Additional Supplementary Files

File Name: Supplementary Movie 1
Description: Time-lapsed animation of a standing wave mode profile of a BGS in a NW GSL with *d* = 140 nm, *e* = 135 nm, and *p* = 420 nm.

File Name: Supplementary Movie 2
Description: Time-lapsed animation of a Mie-coupled BGS propagating from a GSL with *d* = 330 nm, *e* = 315 nm, and *p* = 800 nm into a NW WG.

File Name: Supplementary Movie 3
Description: Time-lapsed animation of a mode profile in a NW GSL with *d* = 140 nm, *e* = 115 nm, and *p* = 400 nm excited by a Gaussian light with FWHM = 7 µm.

File Name: Supplementary Movie 4
Description: Time-lapsed animation of a mode profile in a NW GSL with *d* = 140 nm, *e* = 115 nm, and *p* = 400 nm excited by a Gaussian light with FWHM = 700 nm.
